# Ongoing rubella epidemic in Osaka, Japan, in 2018–2019

**DOI:** 10.5365/wpsar.2019.10.3.001

**Published:** 2020-06-30

**Authors:** Daiki Kanbayashi, Takako Kurata, Hideyuki Kubo, Atsushi Kaida, Seiji P Yamamoto, Kazutaka Egawa, Yuki Hirai, Kazuma Okada, Ryo Ikemori, Takahiro Yumisashi, Akira Yamamoto, Hideki Yoshida, Takanori Hirayama, Kazuyoshi Ikuta, Kazushi Motomura

**Affiliations:** a Osaka Institute of Public Health, Osaka, Japan.; b Sakai City Institute of Public Health, Osaka, Japan.; c Osaka City Health Center, Osaka, Japan.; d Osaka Prefectural Government, Osaka, Japan.; § Both authors contributed equally to this work.

Rubella is a typically mild contagious disease caused by the rubella virus. (*1*) However, when a pregnant woman is infected with rubella virus, fetal death or congenital rubella syndrome (CRS) can occur. (*1*) The number of rubella and CRS cases has been reduced in many countries as a result of rubella vaccinations. (*2*) To prevent the occurrence of CRS, the World Health Organization (WHO) Global Vaccine Action Plan 2011–2020 set the goal of achieving rubella elimination in at least five WHO regions by 2020. (*3*)

In Japan, an estimated 100 000 cases of rubella occurred every year and outbreaks occurred approximately every 5 years until about 1990. With routine immunizations, the scale of the epidemics has been shrinking and the length of time between epidemics has been growing longer. The last outbreak occurred in 2012–2013, with more than 17 000 cases of rubella and 45 cases of CRS. (*4*) From 2013 to mid-2018, only sporadic or imported cases of rubella were reported in Japan. (*4*, *5*) However, an upsurge of rubella cases was observed between July and August 2018 in the south Kanto region (Chiba, Kanagawa and Tokyo prefectures), and epidemics were subsequently reported in regions of Japan. (*6*) In 2018, 2917 cases of rubella were reported, marking the second largest epidemic since 2008, when rubella was classified as a notifiable disease in Japan. (*6*) During the first half 2019, 1935 cases of rubella and three cases of CRS were reported. (*7*) The characteristics of rubella epidemics in Osaka prefecture are described in this text. We also speculate about the cause of the nationwide epidemics.

**Figure 1:**
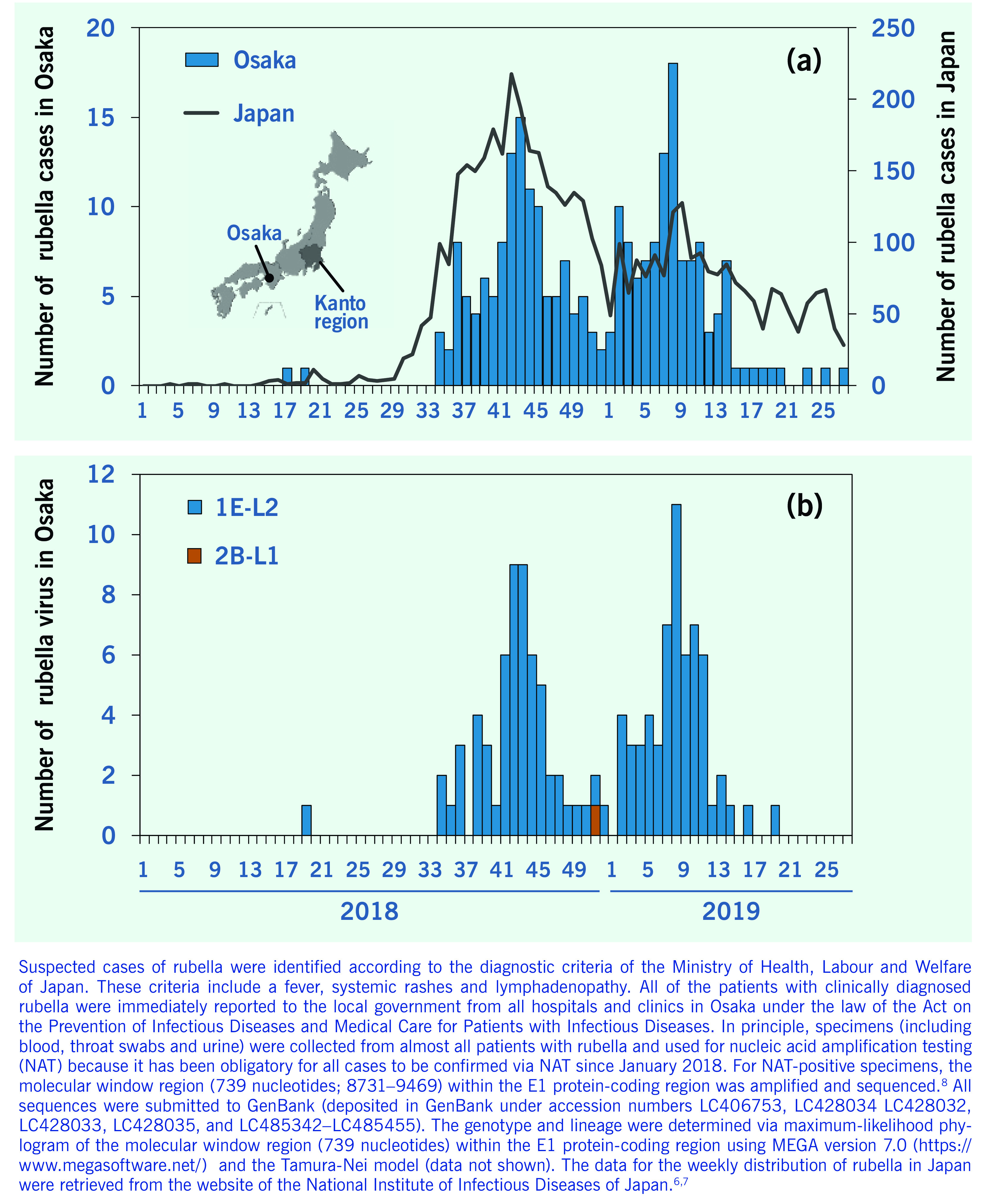
**Weekly distribution of (a) the number of reported rubella cases and (b) the number of detected rubella viruses in 2018–2019**

In total, 123 cases of rubella were reported in 2018 and 118 cases were reported in 2019 (weeks 1–27) (**Fig. 1a**). The first rubella case in Osaka prefecture was reported in week 17 of 2018 (**Fig. 1a**). After the third case was reported in week 34 of 2018, cases of rubella were regularly reported until week 20 of 2019. Among 241 cases reported in 2018–2019, 176 (73.0%) occurred in males. The median patient ages were 40 (range: 1–71) years for males and 32 (range: 0–65) years for females. Vaccination history was unknown in most cases (163 cases, 67.6%), followed by no history of vaccination (57 cases, 23.7%), one dose (18 cases, 7.5%), and two-doses (three cases, 1.2%).

[**insert** Figure 1]

Among the 241 cases reported in 2018–2019, genotypes could be determined in 119 cases. Genotypes were classified as genotype 1E (118/119; 99.2%) and 2B (1/119; 0.8%) (**Fig. 1b**). All genotype 1E strains belonged to genotype 1E lineage 2, and the genotype 2B strain belonged to genotype 2B lineage 1 (**Fig. 1b**). (*8*) All genotype 1E strains detected after week 34 of 2018 in Osaka prefecture were closely related to each other with 99.2–100% nucleotide identity and the representative strains detected before and after week 34 in the Kanto region (accession numbers: LC466969, LC422203, LC422829, LC422204 and LC422205) with 100% nucleotide identity.

The rubella epidemic in Osaka prefecture was part of a large ongoing epidemic of rubella across Japan. Most patients were adult males born on or before 1 April 1979, who had not been targeted for routine rubella immunization during childhood, and males and females born on or after 2 April 1979 with low vaccination coverage. After the 2012–2013 epidemic, the seropositive proportion (haemagglutination-inhibition antibody titre ≥ 1:8) of the total population remained steady at 91.0% (5148/5656). However, among males in their 30s to 50s the seropositive proportion was 84.2% (974/1157) in 2017, in line with that observed before the 2012–2013 epidemic in Japan. (*9*) Therefore, insufficient vaccine coverage may have created a situation in which a new epidemic of rubella emerged in Japan when rubella virus was imported.

The 2012–2013 epidemic was caused by rubella virus strains with a variety of genetic backgrounds, suggesting that these strains were introduced from multiple sources. (*8*) In contrast, the 2018–2019 epidemic was mainly caused by rubella virus strains with the same or very close genetic background. It is unclear whether the 2018–2019 rubella epidemic was caused by the expansion from a single source or several sources in Kanto region. This is because the epidemiological link of most cases is unclear, which is a limitation of the current study. The number of rubella cases related to importation from South-eastern and East Asia doubled in Japan in 2018, compared with the number over the past four years. (*10*)

We believe that the epidemic may be in part attributable to immunization strategies that left a susceptible population in Japan as well as potential introduction of rubella virus from other countries. Although the WHO position paper on rubella vaccines, published in July 2011, stated that the effect of a selective immunization policy is limited, (*11*) the current outbreak highlights that high vaccination coverage with two doses of a rubella-containing vaccine targeting children as well as adults who are hard-to-reach and vulnerable is needed to eliminate rubella. The Ministry of Health, Labour and Welfare of Japan began subsidizing antibody testing and vaccination costs for 16.1 million adult males to raise the vaccine coverage, as indicated by rubella antibody seropositivity of the target generation to at least 90% by the end of 2021. The lessons learnt from this outbreak can be of value to achieve rubella elimination for other countries that have introduced or have planned selective immunization policies.
